# King Edward's Hospital Fund

**Published:** 1913-12-20

**Authors:** 


### The Hospital

**The Workers' Newspaper of Administrative Medicine and Institutional Life, Administration, National Insurance and Health.**

No. 1434, Vol. LV. SATURDAY, DECEMBER 20, 1913. PRICE ONE PENNY.

### KING EDWARD'S HOSPITAL FUND.

The meeting of the Council of this Fund at St. James's Palace on the 13th instant should be memorable in the history of voluntary hospitals. King Edward's Fund was established in consequence of the inability of the existing central funds to raise, individually or collectively, an annual sum large enough to meet the ever-increasing needs of the metropolitan hospitals. At the time it was instituted (1897) the voluntary hospitals showed an aggregate net deficiency of quite £100,000 a year, and all efforts to bridge this abyss during the ten years ending 1895 proved ineffectual. When the Diamond Jubilee of Queen Victoria approached, a multitude of schemes were propounded. But Queen Victoria left the decision to the Prince of Wales, who put forward, with Her Majesty's warm sympathy, that for the creation of what is now known as King Edward VII.'s Hospital Fund for London.

It is interesting to recall that the proposal met with a mixed reception. Thus *The Times* declared the scheme to be worthy of the Prince of Wales and the occasion which had called it into being, and the *Daily Mail* said its details were eminently plain and businesslike, whilst its leading idea had the simplicity of genius. But a weekly review defined it as the "most Utopian and wildest of schemes, thoroughly impracticable, and the work of bad advisers. It would do little or nothing to place the hospitals on a sound financial basis or to raise them in the estimation of the public." Other critics maintained that "its success would be merely temporary," whilst it would "diminish the receipts of existing funds and of the hospitals themselves." It is intensely encouraging to find from the results to-day, that although the task has not been easy the effects of the King's Fund and its results have been remarkable and far-reaching for good.

Financially, the results of the King's Fund have been supremely satisfactory. In sixteen years it has raised and distributed directly amongst the hospitals and convalescent homes, upwards of one million and three-quarter pounds sterling. The total of the invested funds at the end of 1912 amounted to £1,800,000, of which over £1,000,000 represented gifts to capital. The League of Mercy has raised for the King's Fund in fourteen years upwards of £200,000. These results have been obtained without making any appeal to the public which has in any way interfered with the raising of money for voluntary hospitals and kindred institutions, on the lines pursued by them, before the King's Fund was established. Year by year the ordinary income of the London hospitals has shown a steady increase over the previous year, with the exception of 1909, when the aggregate difference in the case of 106 hospitals amounted that year to a total diminution of £14,546, as compared with the year 1908. The increase in the ordinary income of the London hospitals in a single year has been as great as £65,000, and as small as £18,460. But the ordinary income of these hospitals has steadily and continuously increased year by year, with the single exception mentioned. But the King's Fund has exercised a strong influence for good over the whole voluntary system. Thus in sixteen years the total income of British voluntary hospitals has increased by 66 per cent., or in round figures by a total represented by one million and three-quarter pounds per annum. The more closely the figures are studied the more encouraging and wonderful will the financial results since 1897 be found to be.

In addition to its remarkable financial progress, the King's Fund has secured the willing co-operation of the managers of the voluntary hospitals. Indeed, it at present forms the centre of influence amongst them. It has exercised a marked influence for good on the economy of management by its annual statistical report on hospital expenditure, the issue of which has been followed by savings estimated at £47,000 a year. It has promoted the revision of the uniform system of hospital accounts, secured precautions against fire, and done service in matters like the admission of out-patients, the expenses of charity entertainments, and the financial relations of hospitals and medical schools. It is now the recognised centre for the solution of difficult hospital problems, having held several conferences, obtained expert reports, and appointed committees of inquiry. In a word, the King's Fund has become, admittedly, a triumphant success.

But apart from the administrative and financial advantages which the establishment of the King's Fund has brought in its train, we owe other material matters to its institution. The doctrine of personal service was first brought prominently to the public notice, and readily adopted by thousands of people, through the League of Mercy. Amongst the men who first found a place upon the council were several who were believers in personal service, and had made it the practice of their lives. One of these men was the late Sir Julius Wernher, who steadily and quietly worked for the extension of the King's Fund, and who has now put the crown upon his service for the sick by leaving to the Fund one-twelfth of his fortune, which is estimated to yield £450,000. Sir Julius Wernher and his wife have long been active and successful workers for the League of Mercy, and their example has been fruitful in results. We rejoice to know that the principle of personal service, like the voluntary principle, is becoming deep seated in the hearts of the younger generation. We have confidence therefore in declaring that our voluntary hospitals in the financial sense rest upon a sure foundation.

